# Overview of heteroresistance to multiple antibiotics in clinical *Klebsiella pneumoniae* isolates and combination therapeutic strategies

**DOI:** 10.1093/jacamr/dlaf071

**Published:** 2025-05-13

**Authors:** Qiaoyu Zhang, Lirong Wen, Shanshan Li, Linwen Zheng, Yuli Nie, Jiansen Chen

**Affiliations:** Department of Nosocomial Infection Control, Fujian Medical University Union Hospital, Fuzhou, Fujian 350001, China; Department of Nosocomial Infection Control, Fujian Medical University Union Hospital, Fuzhou, Fujian 350001, China; Department of Nosocomial Infection Control, Fujian Medical University Union Hospital, Fuzhou, Fujian 350001, China; Department of Nosocomial Infection Control, Fujian Medical University Union Hospital, Fuzhou, Fujian 350001, China; Department of Nosocomial Infection Control, Fujian Medical University Union Hospital, Fuzhou, Fujian 350001, China; Department of Nosocomial Infection Control, Fujian Medical University Union Hospital, Fuzhou, Fujian 350001, China

## Abstract

**Objectives:**

To assess the prevalence of heteroresistance in 201 clinical isolates of *Klebsiella pneumoniae* to 16 clinically significant antibiotics. Furthermore, to investigate the interaction effects of combination antibiotic therapies for heteroresistant isolates.

**Methods:**

Isolates were pre-screened for growth of resistant subpopulations at resistant breakpoint concentrations for each isolate/antibiotic combination. Any strain containing colony growth at the resistant breakpoint was selected as a candidate heteroresistant strain, and population analysis profiling (PAP) tested for confirmation of HR phenotype. Dual PAP and time–kill assay were conducted to assess the efficacy of antibiotic combinations in suppressing resistant subpopulations.

**Results:**

Ninety-seven percent of isolates were shown to be heteroresistant to at least one antibiotic. Heteroresistance to at least two antibiotics was exhibited by 72.1% of strains. The prevalence of heteroresistance varied across antibiotics, with proportions ranging from 1.5% for imipenem to 85.1% for polymyxin B. The case of Kp486 was heteroresistant to amikacin, ceftazidime/avibactam, tigecycline and polymyxin B. The resistant subpopulations displayed distinct PAP curves and differences in growth and killing kinetics, indicating independent mechanisms for heteroresistance to each of the four antibiotics. Dual PAP experiments showed enhanced killing effects for combinations of antibiotics. In time–kill experiments, pairwise combinations of four drugs achieved a reduction of 3 to 6 logs within 6 h, preventing regrowth of resistant subpopulations. However, combinations with ampicillin did not enhance the activity of tigecycline, polymyxin B or ceftazidime/avibactam.

**Conclusions:**

Heteroresistance in clinical *K. pneumoniae* is common and can complicate treatment outcomes. The effects of combination antibiotic therapy depend on the heteroresistance of bacteria to both drugs.

## Introduction

Unlike homogeneity, ‘heteroresistance’ refers to a phenomenon in which genetically identical bacterial subgroups exhibit varying levels of susceptibility to a specific antibiotic, despite having similar overall genetic profiles. Heteroresistance can be defined in several ways, but the most typical and clinically significant form is when the majority of the bacterial population is susceptible to antibiotics, while a small subset contains highly resistant subpopulations.^1^ Current research predominantly concentrates on this scenario. The clinical breakpoint is the specific antibiotic concentration at which bacterial growth is associated with clinical resistance, while growth inhibition at this concentration is linked to clinical susceptibility and successful treatment outcomes. Conventional susceptibility testing usually determines antibiotic therapy based on breakpoints for bacteria. For heteroresistant strains, however, conventional susceptibility testing may inadvertently favour the growth of resistant subpopulations, potentially resulting in treatment failure.^2,3^

*Klebsiella pneumoniae* is a common pathogen in hospitals that causes infections. MDR *K. pneumoniae*, especially carbapenem-resistant strains, poses a significant threat to patient diagnosis and treatment. Recently, heteroresistance has been widely recognized to exist across different antibiotics used in treating *K. pneumoniae* infections.^4–7^ However, there is still limited overall understanding of the heteroresistance to multiple antibiotics in clinical *K. pneumoniae* isolates.

In this study, we only focus on isolates that are susceptible to at least one antibiotic by conventional susceptibility testing but contain highly resistant subpopulations. We collected 201 strains of *K. pneumoniae* from a large tertiary hospital. We used a method combining rapid screening with a classical experiment, population analysis profiling (PAP), to screen for heteroresistance to 16 commonly used antibiotics. The effect of combination therapy was explored by time–kill assay *in vitro*. Understanding and paying attention to the presence of heteroresistance in *K. pneumoniae* infections is essential for selecting effective treatment strategies. In such cases, combination therapy could be a promising approach, significantly enhancing treatment outcomes and minimizing the risk of failure.

## Materials and methods

### Bacterial isolates and clinical susceptibility testing

Two hundred and one clinical isolates of *K. pneumoniae* [104 carbapenem-resistant *K. pneumoniae* (CR-Kp) and 97 carbapenem-susceptible *K. pneumoniae* (CS-Kp)] were collected during 2018 from Fujian Medical University Union Hospital, a tertiary care teaching hospital with 3500 beds located in south-east China (Table S1, available as Supplementary data at *JAC-AMR* Online). Bacteria were streaked onto Mueller–Hinton agar (MHA) plates (Oxoid, Basingstoke, UK) from frozen glycerol stocks. The strains were stored in LB medium (Thermo Fisher Scientific, Shanghai, China) containing 15% glycerol at −80°C. The VITEK 2 Compact system (bioMérieux, Marcy l’Étoile, France) was used for identification of bacterial species. Determination of the MIC of different antibiotics was performed by the broth microdilution method. *Escherichia coli* ATCC 25922 was used as a control strain for the broth microdilution method.

### Pre-screening of clinical isolates

**Figure 1.**
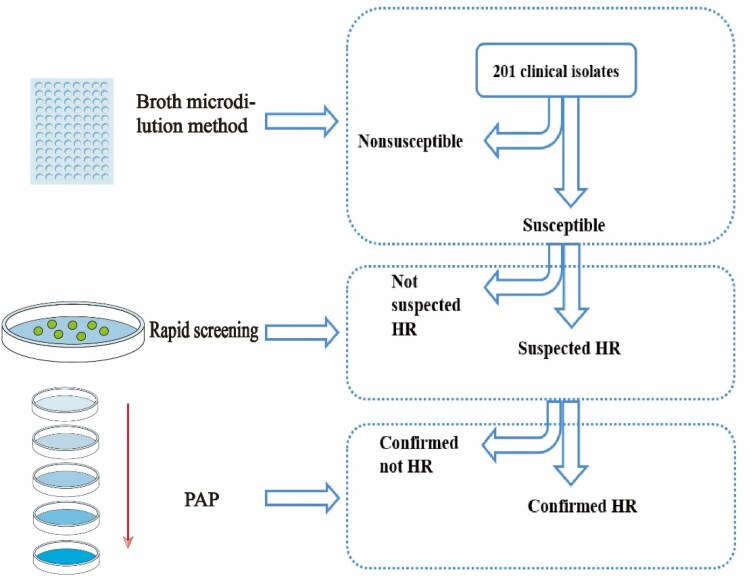
Flowchart of heteroresistant *K. pneumoniae* rapid screening and confirmation. The broth dilution method was used to determinate the antibiotic MIC values. The plate containing antibiotic at its resistant breakpoint concentration was used to select heteroresistance candidates. Moreover, PAP was conducted to confirm the heteroresistance of the isolates.

All clinical isolates were pre-screened for growth of resistant subpopulations at resistant breakpoint concentrations for each isolate/antibiotic combination (Figure 1). Briefly, approximately 10^8^ cfu from the overnight cultures were plated on MHA plates containing antibiotics at the resistant breakpoint of each antibiotic. After incubation at 37°C for 24 h, any strain growing colonies on the plate (>1 × 10^−7^ subpopulation) was selected as a candidate heteroresistant strain and PAP tested for confirmation of HR phenotype. Two biological replicates were used for each isolate.

### PAP

The PAP method was employed to detect heteroresistance and assess the proportion of surviving bacteria under the influence of 16 different antibiotics. It was conducted as described previously,^4,8^ with some modifications. Briefly, solid agar plates were made for each antibiotic at six concentrations containing 0, 0.125, 0.25, 0.5, 1, 2 and 4 times the breakpoint, using MHA. Breakpoint concentrations for Enterobacteriaceae from CLSI [ampicillin 32 mg/L, cefazolin 8 mg/L, ceftazidime 16 mg/L, cefepime 16 mg/L, aztreonam 16 mg/L, imipenem 4 mg/L, meropenem 4 mg/L, amoxicillin/clavulanate 32/16 mg/L, piperacillin/tazobactam 32/4 mg/L, cefoperazone/sulbactam 64 mg/L, ceftazidime/avibactam 16/4 mg/L, amikacin 16 mg/L, ciprofloxacin 1 mg/L, fosfomycin 256 mg/L (+25 mg/L glucose-6-phosphate (G6P)], FDA (tigecycline 8 mg/L) or EUCAST (polymyxin B 4 mg/L) were used (Table S2). Dual PAP experiments were performed similarly with six concentrations of antibiotics tested, with both drugs in each plate at the same multiple of their respective breakpoints (0, 0.125, 0.25, 0.5, 1, 2 and 4 times the resistant breakpoint). Isolates to be tested were grown overnight in Mueller–Hinton broth. Serial dilutions from 10^8^ to 10^2^ cfu were plated at each concentration of antibiotic. Colonies were enumerated after 24 h growth at 37°C. Two biological replicates were used for each isolate. The detection limit of antibiotic-resistant subpopulations was 20 cfu/mL. An isolate was classified as heteroresistant when a high-frequency subpopulation (>1 × 10^−7^) of cells could grow at a concentration of drugs at least at the resistant breakpoint of each antibiotic.^9^

### Time–kill assays

Time–kill assays were conducted by inoculating 10^6^ cfu/mL of the culture into CAMHB with and without antibiotic(s). The resistant breakpoint concentrations were used. Cultures were incubated at 37°C, shaking at 200 rpm, for 1–24 h. cfu were determined by serial diluting bacteria in PBS prior to plating on MHA plates at various timepoints in the assay (0, 2, 4, 6, 8, 12, 18 and 24 h timepoints were used for time–kill assays). The limit of antibiotic-resistant subpopulations was 20 cfu/mL. All experiments were performed in triplicate.

### Statistical analysis

All the data presented are based on measurements taken from separate samples. To ensure reproducibility, all experiments were conducted at least twice. Statistical analysis and plotting were performed using GraphPad Prism software.

## Results

### An overview of heteroresistance in clinical *Klebsiella pneumoniae* isolates

Heteroresistance (HR) has been reported in multiple bacterial species and with various antibiotics. However, it is still unclear whether multidrug heteroresistance is a rare phenomenon or a common occurrence in clinical isolates of *Klebsiella pneumoniae*. The PAP method was employed to detect heteroresistance and assess the proportion of surviving bacteria under the influence of 16 different antibiotics. All susceptible isolates were first screened for the growth of resistant subpopulations at resistant breakpoint concentrations, and the presence of heteroresistance was then confirmed using the PAP test (Figure 1 and Table S2).

**Figure 2.**
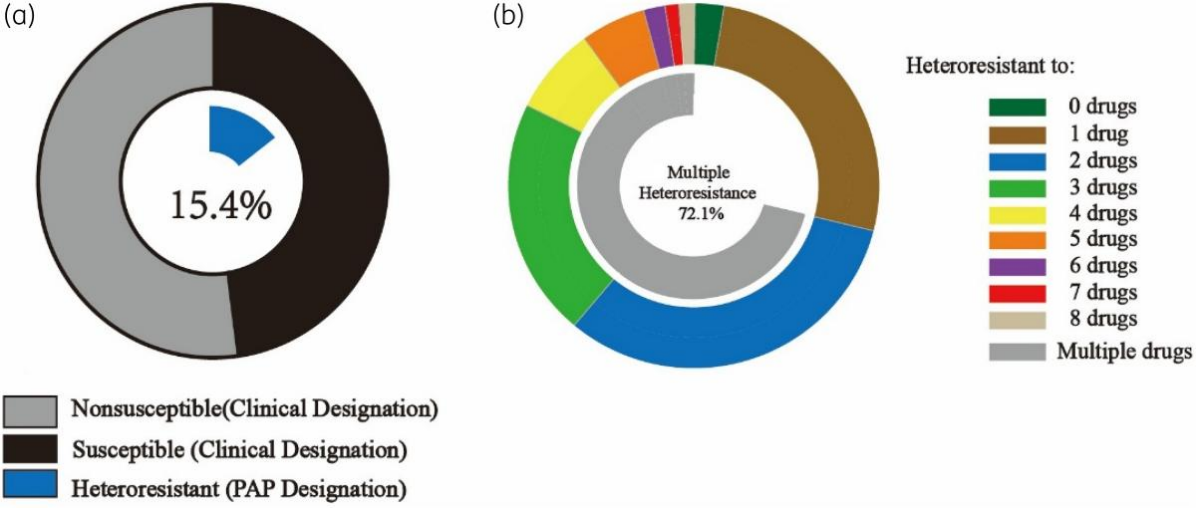
Multiple heteroresistance is common in clinical isolates. (a) Percentage of clinical susceptibility testing results (for 201 isolates and 16 antibiotics) classified as non-susceptible (light grey) or susceptible (black). Those designated as heteroresistant by PAP are indicated by the central blue ring. (b) Isolates classified by the number of antibiotics to which they are heteroresistant out of the 16 tested (none was heteroresistant to more than 9) with the percentage heteroresistant to more than one antibiotic indicated by central grey ring.

**Figure 3.**
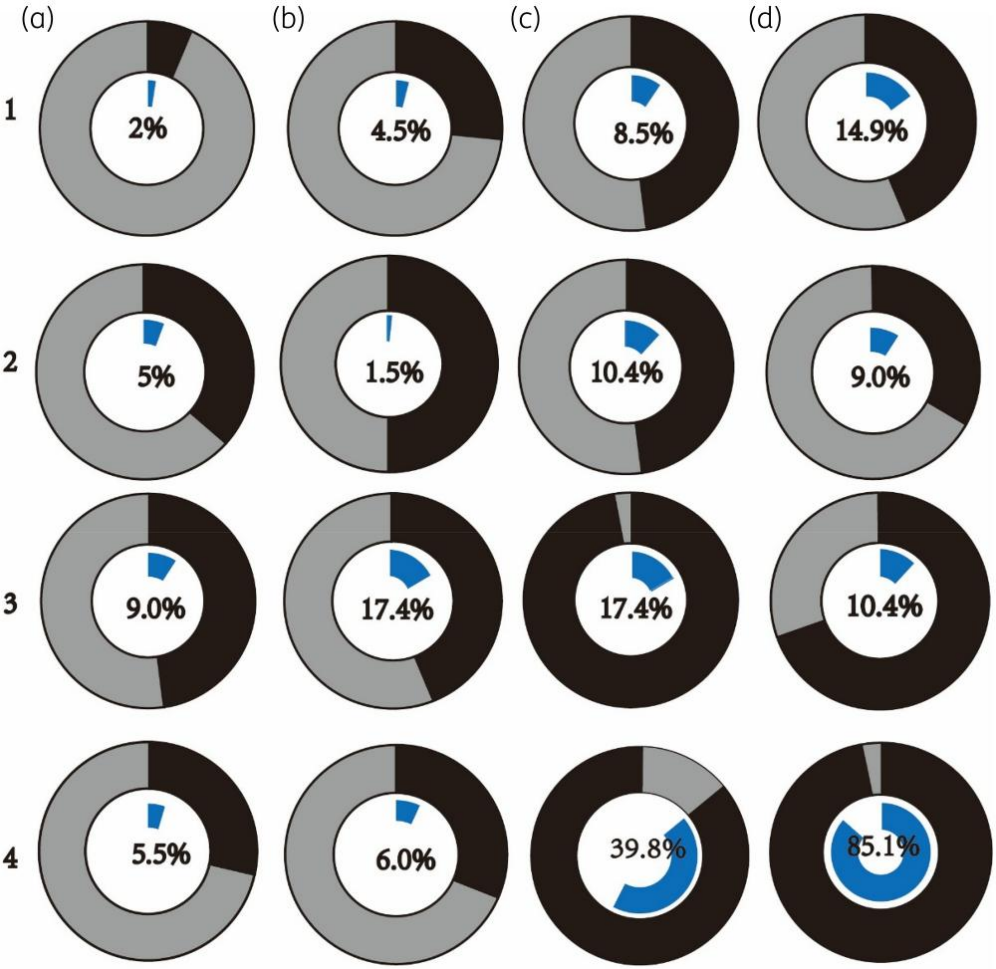
Percentage of heteroresistance to 16 antibiotics. A1–D4, 201 *K. pneumoniae* clinical isolates were screened for heteroresistance to 16 antibiotics and confirmed by the PAP method. Percentage of clinical susceptibility testing results classified as non-susceptible (light grey) or susceptible (black). Those designated heteroresistant by PAP are indicated by the central blue ring. Percentages of isolates heteroresistant to each antibiotic are shown for: a1, ampicillin; b1, cefazolin; c1, ceftazidime; d1, cefepime; a2, aztreonam; b2, imipenem; c2, meropenem; d2, amoxicillin/clavulanate; a3, piperacillin/tazobactam; b3, cefoperazone/sulbactam; c3, ceftazidime/avibactam; d3, amikacin; a4, ciprofloxacin; b4, fosfomycin; c4, tigecycline; d4, polymyxin B.

**Figure 4.**
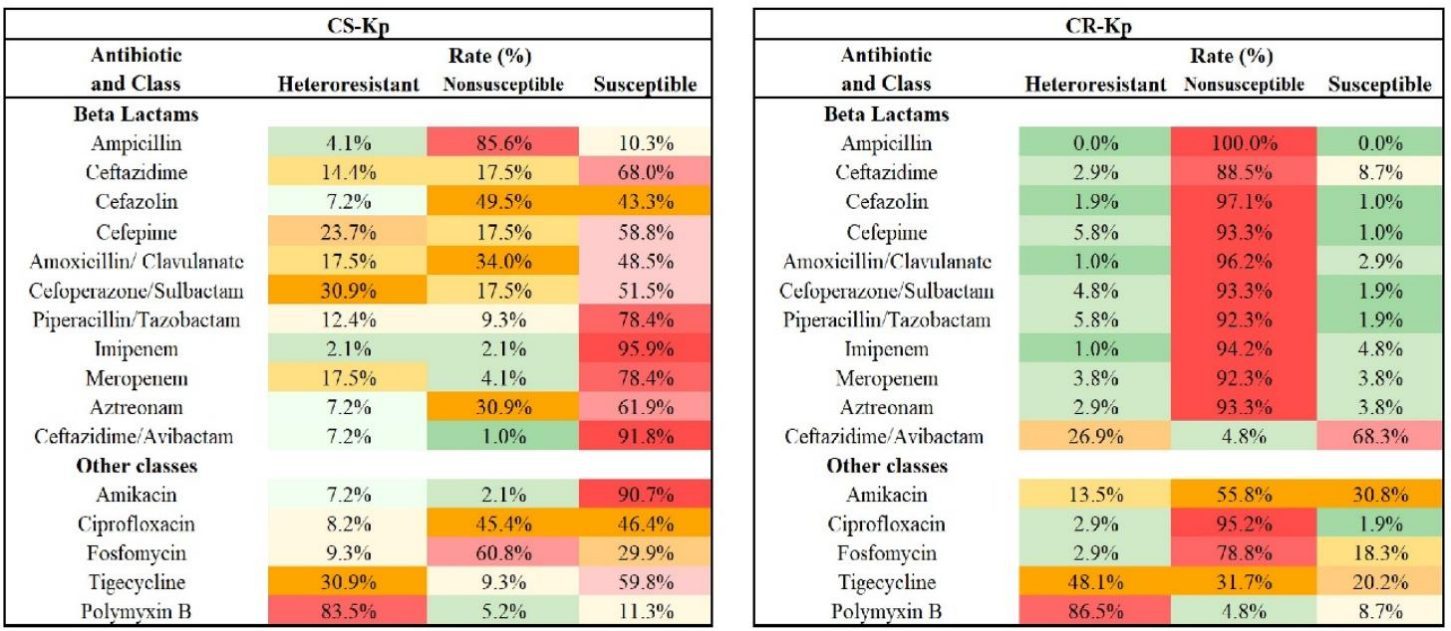
Antibiotic susceptibility of CR-Kp or CS-Kp isolates as designated by PAP. Frequency of susceptibility, heteroresistance and non-susceptibility of the 104 CR-Kp and 97 CS-Kp isolates to each of the indicated antibiotics as determined by PAP. Red shading indicates a higher proportion while green shading indicates a lower proportion.

We found all the strains that survived on the plate containing antibiotic at the concentration of the resistant breakpoint could be confirmed by PAP testing. Ninety-seven percent of isolates showed heteroresistance to at least one of the 16 antibiotics tested (Figure 2 and Table S3) and 72.1% of strains exhibited heteroresistance to at least two drugs (Figure 2 and Table S3). Each tested antibiotic exhibited heteroresistance, with widely varying proportions across different drugs, ranging from 1.5% for imipenem to 85.1% for polymyxin B (Figure 3). When comparing 104 CR-Kp and 97 CS-Kp strains, we found that while CS-Kp strains generally exhibited low resistance to most antibiotics (except ampicillin and fosfomycin), a significant portion of susceptible drugs showed heteroresistance (Figure 4). In addition, compared with CS-Kp, CR-Kp exhibited higher rates of heteroresistance to antibiotics considered as the last line of defence against superbugs, such as ceftazidime/avibactam (26.9% versus 7.2%), tigecycline (48.1% versus 30.9%) and polymyxin B (86.5% versus 83.5%) (Figure 4). This significantly increases the difficulty in treating CR-Kp infections.

### Clinical isolate Kp486 exhibits distinct heteroresistance to four antibiotics

**Figure 5.**
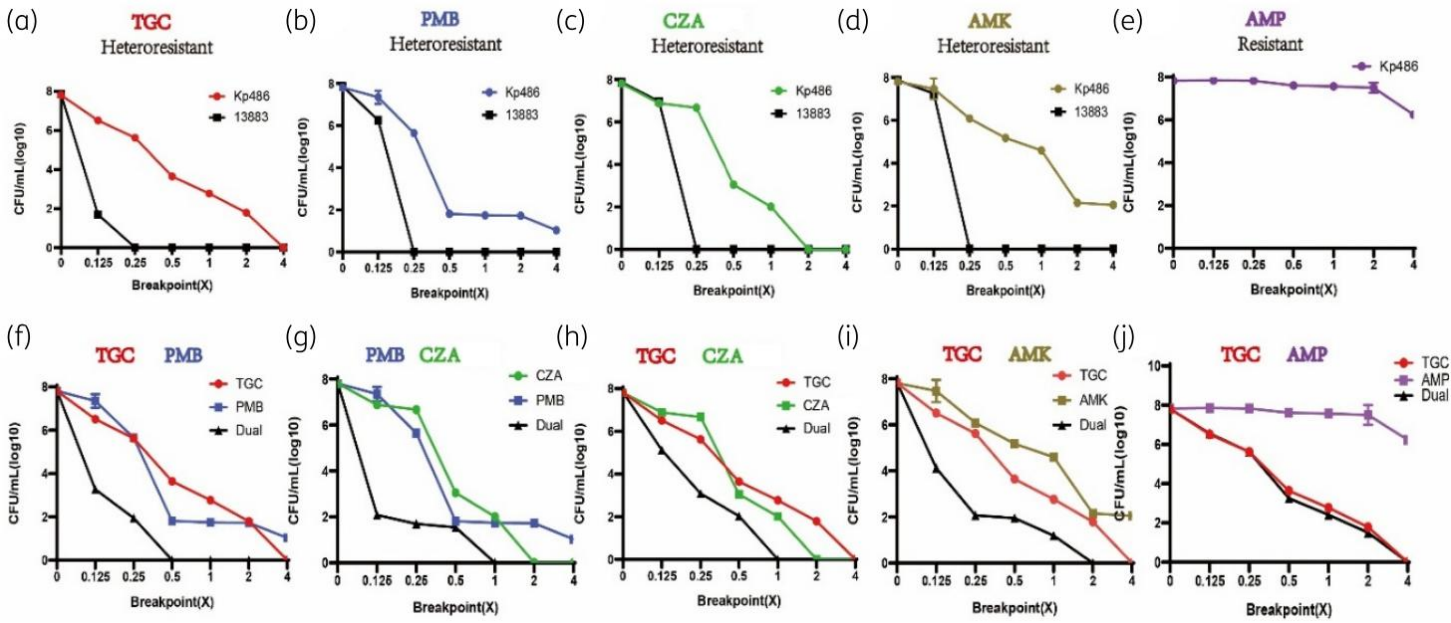
PAP confirmation of carbapenem-resistant clinical *K. pneumoniae* Kp486. PAP of Kp486 and control susceptible strains plated on the indicated antibiotic at gradient concentration relative to its breakpoint of (a) tigecycline (TGC, 8 mg/L); (b) polymyxin B (PMB, 4 mg/L); (c) ceftazidime/avibactam (CZA, 16/4 mg/L); (d) amikacin (AMK, 16 mg/L); (e) ampicillin (AMP, 32 mg/L). (f) Kp486 was treated with a combination of TGC and PMB; (g) PMB and CZA; (h) TGC and CZA; (i) TGC and AMK; or (j) TGC and AMP. Error bars indicate standard deviations of triple repeated experiments.

In this study, we found some CR-Kp isolates were resistant to nearly all available antibiotics except for some distinct antibiotics such as the last-line antibiotics. There is a lack of therapeutic options to treat such infections. The phenomenon of heteroresistance to these antibiotics may enhance the difficulty of clinical treatment. However, effective infection control has been achieved using antibiotic combinations that leverage heteroresistance to multiple drugs.^3,8^ We identified a clinical isolate CR-Kp Kp486 as resistant to multiple antibiotics and exhibiting heteroresistance to four antibiotics involved the last-line antibiotics (amikacin, ceftazidime/avibactam, tigecycline and polymyxin B) (Figure 5 and Table S4).

Approximately 5 logs of Kp486 cells were killed by tigecycline concentrations below the clinical breakpoint (MIC = 8 mg/L) (Figure 5a). However, the resistant subpopulations specifically survived in the presence of tigecycline concentrations exceeding the breakpoint (Figure 5a). PAP testing showed that the tigecycline-resistant subpopulation survived at a concentration 2-fold higher than the breakpoint. In contrast, all cells of the susceptible isolate ATCC 13883 were eradicated at a tigecycline concentration below the breakpoint (Figure 5a). PAP testing with other antibiotics revealed that Kp486 also displayed heteroresistance to three additional antibiotics: polymyxin B, ceftazidime/avibactam and amikacin (Figure 5b–d). For both polymyxin B (Figure 5b) and amikacin (Figure 5d), a resistant subpopulation survived at concentrations 4-fold higher than the breakpoint. For ceftazidime/avibactam, the resistant subpopulation survived at 2-fold the breakpoint (Figure 5c). In contrast, Kp486 showed homogeneous resistance to ampicillin, as evidenced by the absence of bacterial killing at any of the tested concentrations (Figure 5e).

### Clinical isolate Kp486 could be killed by combinations of heteroresistant antibiotics

**Figure 6.**
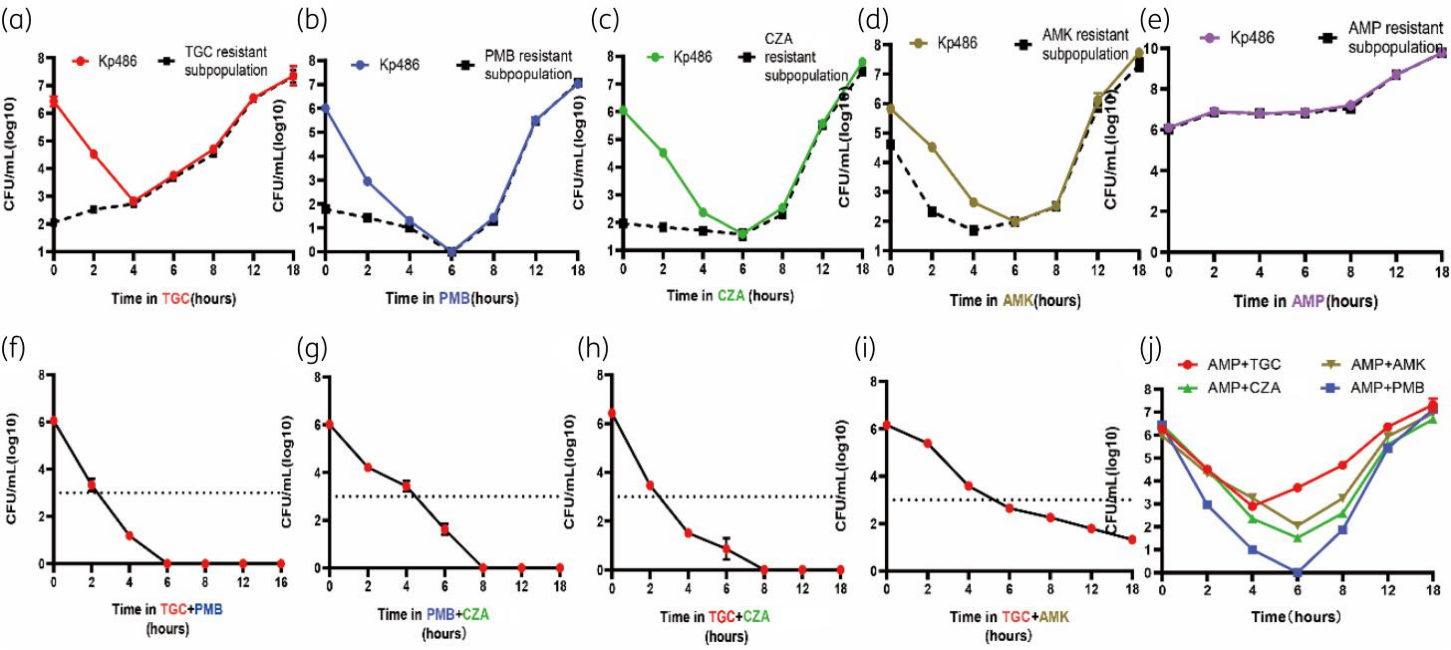
Time–kill curves for antibiotic monotherapy and combination treatment against carbapenem-resistant clinical *K. pneumoniae* Kp486. (a) Kp486 was treated with TGC (8 mg/L); (b) PMB (4 mg/L); (c) CZA (16/4 mg/L); (d) AMK (16 mg/L) or (e) AMP (32 mg/L) at their breakpoint concentrations. Bacteria were plated at the indicated timepoints for counting the total cfu (solid line) and the homologous resistant (dashed line) cfu. (f) Kp486 was treated with a combination of TGC (8 mg/L) and PMB (4 mg/L); (g) PMB (4 mg/L) and CZA (16/4 mg/L); (h) TGC (8 mg/L) and CZA (16/4 mg/L); (i) TGC (8 mg/L) and AMK (16 mg/L); or (j) four antibiotics with AMP (32 mg/L). Error bars indicate standard deviations of triple repeated experiments.

We tracked the growth kinetics of Kp486 in the presence of tigecycline, polymyxin B, ceftazidime/avibactam and amikacin (Figure 6). Serially diluted bacteria were also plated on MHA plates with antibiotic added (tigecycline 8 mg/L, polymyxin B 4 mg/L, ceftazidime/avibactam16/4 mg/L, amikacin 16 mg/L and ampicillin 32 mg/L) (Figure 6a–e). We found that after an initial phase of killing susceptible cells, the resistant subpopulation rapidly proliferated in response to each antibiotic (Figure 6a–d). In contrast, exposure to ampicillin did not result in the initial bacterial reduction observed with the other antibiotics, indicating that Kp486 exhibits homogeneous resistance to ampicillin (Figure 6e). The tigecycline-, polymyxin B-, ceftazidime/avibactam- and amikacin-resistant subpopulations displayed differing PAP curves (Figure 5a–d) and kinetics of killing and growth (Figure 6a–d) in their respective antibiotics, suggesting there are independent mechanisms responsible for four antibiotics’ heteroresistance in CR-Kp. Therefore, we hypothesized that resistant subpopulations could survive antibiotic pressure from these antibiotics but were killed by the combination. Indeed, the use of dual drug PAP (‘dual PAP’)—which involved increasing concentrations of two antibiotics, each at a multiple of their respective breakpoints—demonstrated that the killing effect of each dual antibiotic combination was enhanced (Figure 5f–i). For Kp486, resistant subpopulations survived at 2-fold the resistant breakpoint of tigecycline and 4-fold that of polymyxin B (Figure 5a and b). However, these subpopulations could be completely inhibited by their combination at 0.5-fold the breakpoint (Figure 5f). Similarly, for these four antibiotics exhibiting heteroresistance, pairwise combinations were more effective in killing the heteroresistant subpopulations compared with using a single antibiotic alone. This indicated that killing was additive. In a time–kill experiment, the tigecycline/polymyxin B, tigecycline/ceftazidime/avibactam, ceftazidime/avibactam/polymyxin B and tigecycline/amikacin combinations each had 3–6 logs of killing within 6 h (Figure 6f–i), preventing the regrowth of resistant subpopulations (Figure 6a–d). However, in both dual PAP (Figure 5) and time–kill experiments (Figure 6), the activity of amikacin, tigecycline, polymyxin B or ceftazidime/avibactam was not enhanced by combination with ampicillin (Figures 5j and 6j). This suggests that the effects of combination antibiotic therapy depend on the heteroresistance of bacteria to both drugs.

## Discussion

Antibiotic heteroresistance refers to a heterogeneous strain in which subpopulations display higher levels of antibiotic resistance than the predominant population.^1^ Previous studies have shown that approximately half of carbapenem-resistant Enterobacterales (CRE) were heteroresistant to some antibiotics, such as tigecycline, polymyxin B, meropenem, doripenem and cefepime. These cases involved a small proportion of resistant cells, which were typically not detected by current clinical susceptibility testing methods.^8,10,11^ The small resistant subpopulations were specifically enriched in the presence of antibiotics both *in vivo* and *in vitro.*^12,13^ In our study, under antibiotic pressure, the resistant subpopulations gradually became predominant after the later incubation period (Figure 6a–d). This suggests that heteroresistance may be the early stages for bacteria changing from susceptible to resistant and indicated that the potential development of resistance may be missed. Heteroresistance is widely recognized as a key factor in treatment failure.^5,13–15^ If conventional monotherapy protocols are used for heteroresistant isolates, this could potentially lead to treatment failure and encourage susceptible strains to evolve into resistant ones. Therefore, detecting and characterizing heteroresistance in clinical isolates is essential to optimize the effectiveness of antibiotics.^3,16^ However, there have been only a few reports considering the use of more than one antibiotic for clinical pathogens. In the present study, we investigated heteroresistance to multiple antibiotics in clinical *K. pneumoniae* isolates belonging to the ESKAPE group.^17^

Detecting heteroresistance through conventional antibiotic susceptibility tests is unreliable and highly dependent on the method employed. Resistant subpopulations in heteroresistant strains may become detectable using disc diffusion and gradient diffusion methods, such as ETEST, where colonies appear within the zone of inhibition.^13^ The gold-standard method to detect heteroresistance is PAP, which consists of labour-intensive agar microdilution with a range of antibiotic concentrations.^18^ Unfortunately, this method is not practical for clinical use due to it being time-consuming and complex. There is a need for new diagnostics that are sensitive enough to detect low-frequency resistant cells while maintaining the high reproducibility and simplicity expected in clinical testing. In this study, we used a method combining rapid screening with a classical experiment (PAP) to screen for heteroresistance of 16 commonly used antibiotics.

The rapid screening method used in our study omits the conventional step of inoculating bacteria on a gradient concentration of drug-containing plates, only retaining the plate containing the specific antibiotic concentration at the resistant breakpoint. This cost-efficient approach significantly saves time in preparing drug-containing plates and inoculation when performing PAP testing. All the strains exhibiting heteroresistance confirmed by PAP testing can be screened using a plate containing the breakpoint concentration, suggesting the high specificity of this rapid screening method in finding heteroresistance in *K. pneumoniae* isolates. Of the clinical *K. pneumoniae* isolates that were determined to be susceptible to 16 antibiotics, 72.1% were identified as multiply heteroresistant (heteroresistant to more than one antibiotic). These data indicate multiple heteroresistance might be prevalent among clinical *K. pneumoniae* isolates, consistent with recent studies by Band *et al.*,^19^ suggesting a high likelihood of treatment failure when relying on traditional laboratory susceptibility results for the pathogens.^20,21^

Furthermore, our finding suggests that for antibiotics used as the last-line antibiotics, such as tigecycline, polymyxin B and ceftazidime/avibactam, although fewer isolates exhibit resistance to them, the proportion of heteroresistant isolates with low-frequency resistant subpopulations was higher in CR-Kp, significantly increasing the difficulty in treating CR-Kp infections. This result is consistent with previous findings,^8,10,22–24^ and may be related to the fact that CR-Kp strains are more frequently exposed to these antibiotics during clinical treatment.

Some carefully managed animal experiments have demonstrated that when heteroresistant bacteria are exposed to a lethal dose (3 × 10^8^ cfu), monotherapy often fails due to the initial presence of a small number of resistant cells that are amplified by the antibiotic and can grow to high numbers in the infected host.^5,13^ Similarly, mathematical modelling of heteroresistance suggests that it could result in treatment failure when monotherapy is used.^25,26^ Using CRE Kp486 as an example in our study, we found tigecycline-, polymyxin B-, ceftazidime/avibactam- and amikacin-resistant subpopulations displayed differing PAP curves and kinetics of killing and growth in their respective antibiotics, suggesting there are independent mechanisms responsible for four-antibiotic heteroresistance in CR-Kp. In the time–kill assays, tigecycline, polymyxin B, ceftazidime/avibactam and amikacin each killed a large proportion of Kp486 cells but were unable to prevent subsequent outgrowth, while combinations of them could display marked killing and prevent the regrowth of resistant subpopulations. This is supported by recent findings that combinations of clinically approved antibiotics might eradicate infections associated with heteroresistant *K. pneumoniae.*^8,12,19,27^ In our previous study, we examined the combined effect of polymyxin B and tigecycline against *K. pneumoniae* isolates exhibiting dual heteroresistance to both antibiotics.^8^ As anticipated, monotherapy with either polymyxin B or tigecycline showed only a transient inhibitory effect. While susceptible bacteria were eliminated, the resistant subpopulations survived and amplified quickly. However, the combination of polymyxin B and tigecycline, when used at reduced doses, successfully eradicated both susceptible and resistant subpopulations. Other studies have also shown that the combination of ceftazidime/avibactam enhanced the efficacy of polymyxin B against heteroresistant carbapenemase-producing *K. pneumoniae* isolates and prevented the emergence of polymyxin B-resistant subpopulations *in vitro.*^12^ Another isolate showed homogeneous resistance to colistin and heteroresistance to fosfomycin and ceftazidime.^19^ Only dual therapy with fosfomycin and ceftazidime was effective in eradicating the infection, while any combination involving colistin (colistin + fosfomycin or colistin + ceftazidime) was ineffective.^19^ In our study, in both dual PAP (Figure 5j) and time–kill experiments (Figure 6j), the activity of tigecycline, polymyxin B, ceftazidime/avibactam or amikacin was not enhanced by combination with ampicillin, which exhibited homogenous resistance. This finding suggests that multidrug therapies should employ antibiotics that target various heteroresistant subpopulations, rather than those aimed at homogeneous resistant populations.

This study shows that heteroresistance to multiple clinically relevant antibiotics is highly prevalent in clinical *K. pneumoniae* isolates. Therefore, further research is urgently needed on heteroresistance detection and its prevalence, the genetic mechanisms underlying heteroresistance, and the potential impact of resistant subpopulations on treatment outcomes. Of importance, the presence of heteroresistance in *K. pneumoniae* should lead to a preference of combination regimens instead of monotherapy in clinical practice, especially in high-inoculum infections. Further studies are needed to clarify the clinical significance of heteroresistance.

## Supplementary Material

dlaf071_Supplementary_Data

## Supplementary data

Tables S1 to S4 are available as Supplementary data at *JAC-AMR* Online.
