# Supplementary material for: Overview of heteroresistance to multiple antibiotics in clinical Klebsiella pneumoniae isolates and combination therapeutic strategies
Source: JAC Antimicrob Resist. 2025 May 13;7(3):dlaf071. doi: 10.1093/jacamr/dlaf071 (PMC12070040; doi:10.1093/jacamr/dlaf071)
Supplement: dlaf071_Supplementary_Data [file dlaf071_supplementary_data.docx]

Supplementary Material

**Supplementary table 1.** *Klebsiella pneumoniae* patient demographics. The wards of patients are indicated, as are culture source, and patient sex and age group.

|  | **Total** | **Rate(%)** |
| --- | --- | --- |
| **Wards** |  |  |
| ICU | 43 | 21.4% |
| IM Ward | 82 | 40.8% |
| Surgical Ward | 76 | 37.8% |
| **Culture Source** |  |  |
| Urine | 29 | 14.4% |
| Blood | 24 | 11.9% |
| Sputum | 106 | 52.7% |
| Drain fluid | 15 | 7.4% |
| Secretion | 27 | 13.4% |
| **Sex** |  |  |
| Male | 127 | 63.2% |
| Female | 74 | 36.8% |
| **Age Group** |  |  |
| 0-9 | 3 | 1.5% |
| 10-19 | 9 | 4.5% |
| 20-29 | 5 | 2.5% |
| 30-39 | 22 | 10.9% |
| 40-49 | 33 | 16.4% |
| 50-59 | 40 | 19.9% |
| 60-69 | 44 | 21.9% |
| 70-79 | 19 | 9.5% |
| 80-89 | 14 | 7.0% |
| 90-99 | 12 | 6.0% |

**Supplementary table 2.** Drug resistant breakpoint and concentration used in PAP.

| **Antibiotic And Class** | **Drug Breakpoint(ug/mL)^1^** | **Drug Concentration for PAP (ug/mL)** | | | | | | |
| --- | --- | --- | --- | --- | --- | --- | --- | --- |
|  |  | **0** | **0.125 folds** | **0.25 folds** | **0.5 folds** | **1 folds** | **2 folds** | **4 folds** |
| **Beta Lactams** |  |  |  |  |  |  |  |  |
| Ampicillin | 32 | 0 | 4 | 8 | 16 | 32 | 64 | 128 |
| Cefazolin | 8 | 0 | 1 | 2 | 4 | 8 | 16 | 32 |
| Ceftazidime | 16 | 0 | 2 | 4 | 8 | 16 | 32 | 64 |
| Cefepime | 16 | 0 | 2 | 4 | 8 | 16 | 32 | 64 |
| Aztreonam | 16 | 0 | 2 | 4 | 8 | 16 | 32 | 64 |
| Imipenem | 4 | 0 | 0.5 | 1 | 2 | 4 | 8 | 16 |
| Meropenem | 4 | 0 | 0.5 | 1 | 2 | 4 | 8 | 16 |
| Amoxicillin/Clavulanate | 32/16 | 0 | 4/2 | 8/4 | 16/8 | 32/16 | 64/32 | 128/64 |
| Piperacillin/Tazobactam | 32/4 | 0 | 4/4 | 8/4 | 16/4 | 32/4 | 64/4 | 128/4 |
| Cefoperazone/Sulbactam^1^ | 64 | 0 | 8 | 16 | 32 | 64 | 128 | 256 |
| Ceftazidime/Avibactam | 16/4 | 0 | 2/4 | 4/4 | 8/4 | 16/4 | 32/4 | 64/4 |
| **Other classes** |  |  |  |  |  |  |  |  |
| Amikacin | 16 | 0 | 2 | 4 | 8 | 16 | 32 | 64 |
| Ciprofloxacin | 1 | 0 | 0.125 | 0.25 | 0.5 | 1 | 2 | 4 |
| Fosfomycin | 256 | 0 | 32 | 64 | 128 | 256 | 512 | 1024 |
| Tigecycline | 8 | 0 | 1 | 2 | 4 | 8 | 16 | 32 |
| Polymyxin B | 4 | 0 | 0.5 | 1 | 2 | 4 | 8 | 16 |

1. The resistant breakpoint for cefoperazone/sulbactam refers to the CLSI breakpoint for cefoperazone.

**Supplementary table 3.** Isolates classified by the number of antibiotics to which they are heteroresistant out of the 16 tested.

| **Number of**  **Heteroresistant Antibiotics** | **Rate(%)** |
| --- | --- |
| 1 | 24.9% |
| 2 | 30.8% |
| 3 | 22.9% |
| 4 | 8.5% |
| 5 | 6.5% |
| 6 | 1.5% |
| 7 | 0.5% |
| 8 | 1.5% |
| 0 | 3.0% |

**Supplementary table 4.** Characterization of the carbapenem resistant strain Kp486 phenotypes.

| **Antibiotics** | **Parental strain  MIC(mg/L)** | **Drug susceptibility testing assessment** | **Highest concentration  of growth in PAP(mg/L)** | **Frequency of subpopulations** | **Subpopulations  MIC(mg/L)** |
| --- | --- | --- | --- | --- | --- |
| **Beta Lactams** |  |  |  |  |  |
| Ampicillin | 32 | R | NA | NA | NA |
| Ceftazidime | 16 | R | NA | NA | NA |
| Cefazolin | 64 | R | NA | NA | NA |
| Cefepime | 64 | R | NA | NA | NA |
| Amoxicillin/Clavulanate | 32/16 | R | NA | NA | NA |
| Cefoperazone/Sulbactam | 64 | R | NA | NA | NA |
| Piperacillin/Tazobactam | 128/4 | R | NA | NA | NA |
| Imipenem | 8 | R | NA | NA | NA |
| Meropenem | 8 | R | NA | NA | NA |
| Aztreonam | 64 | R | NA | NA | NA |
| Ceftazidime/Avibactam | 4/4 | H | 16/4 | 1.6×10^-6^ | 16/4 |
| **Other classes** |  |  |  |  |  |
| Amikacin | 2 | H | 64 | 1.7×10^-6^ | 32 |
| Ciprofloxacin | 8 | R | NA | NA | NA |
| Fosfomycin | 256 | R | NA | NA | NA |
| Tigecycline | 2 | H | 16 | 9.5×10^-7^ | 16 |
| Polymyxin B | 1 | H | 16 | 1.7×10^-7^ | 16 |
